# Reduction of Negative Charge in Mercaptoacetyl-Based Chelators Influences the Biodistribution of Prostate-Specific Membrane Antigen-Targeting Pseudopeptides Labeled with Technetium-99m

**DOI:** 10.1021/acsptsci.5c00428

**Published:** 2025-09-25

**Authors:** Ekaterina Bezverkhniaia, Panagiotis Kanellopoulos, Ulrika Rosenström, Vladimir Tolmachev, Anna Orlova

**Affiliations:** † Department of Medicinal Chemistry, 8097 Uppsala University, 751 23 Uppsala, Sweden; ‡ Research Center for Oncotheranostics, Research School of Chemistry and Applied Biomedical Sciences, Tomsk Polytechnic University, 634009 Tomsk, Russia; § Department of Immunology, Genetics and Pathology, Uppsala University, 752 37 Uppsala, Sweden; ∥ Science for Life Laboratory, Uppsala University, 752 37 Uppsala, Sweden

**Keywords:** prostate cancer, prostate specific membrane antigen, technetium-99m, mercaptoacetyl-based chelator, amino acid chains, BQ0500, BQ0501, BQ0413, single-photon emission computed tomography

## Abstract

Prostate cancer (PCa) is the most common cancer and the second leading cause of death among men worldwide. Significant progress has been made in managing PCa by targeting the prostate-specific membrane antigen (PSMA), which holds great promise for improving the accuracy and effectiveness of diagnosis. Previously, we reported a high-affinity glutamate–urea–lysine (EuK)-based PSMA-targeting tracer, BQ0413, containing the maE_3_ chelator for labeling with technetium-99m for single-photon emission tomography diagnostic imaging. BQ0413 demonstrated efficient tumor targeting in PCa patients with concomitant elevated activity retention in the kidneys, which is typical for EuK-based PSMA-targeting tracers. We hypothesized that a decrease in the tracer’s total negative charge, by substituting negatively charged glutamate residues in the maE_3_ chelator with polar neutral serine, could decrease activity retention in the kidneys. The present study aimed to evaluate the tumor targeting and biodistribution profile of two new PSMA-targeting tracers, BQ0500 (maESE) and BQ0501 (maS_3_), in comparison to BQ0413 (maE_3_). Conjugates were successfully radiolabeled with technetium-99m and characterized in vitro and in vivo. [^99m^Tc]­Tc-BQ0500 and [^99m^Tc]­Tc-BQ0501 demonstrated PSMA-specific binding to PC3-pip cells with picomolar affinity; however, the affinity was 3–5-fold compromised in comparison with the reference [^99m^Tc]­Tc-BQ0413. Full replacement of glutamate residues by serines in [^99m^Tc]­Tc-BQ0501 resulted in an improved overall clearance from normal organs with a moderately increased accumulation of activity in the gastrointestinal tract. [^99m^Tc]­Tc-BQ0501 demonstrated efficient tumor targeting and improved tumor-to-background ratios. These results suggest that chelator modifications, such as charge alteration, play a critical role in improving tumor targeting and pharmacokinetics for EuK-based PSMA-targeting tracers.

## Graphical abstract

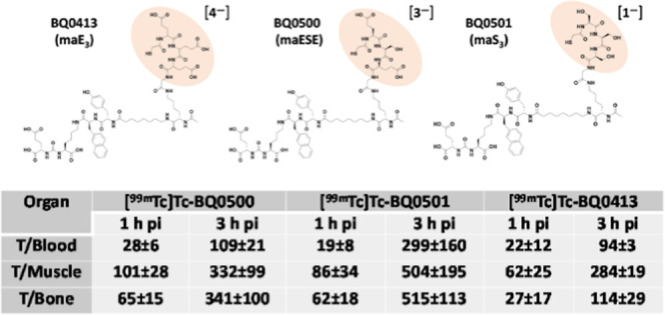

Prostate cancer (PCa) is a biologically and clinically heterogeneous disease, making its diagnosis challenging.^1^ For decades, conventional diagnostic methods such as ultrasound, bone scintigraphy, computed tomography (CT), and magnetic resonance imaging have been routinely used to detect PCa. However, these strategies fail to reveal all PCa cases due to its biological heterogeneity and multifocality.^2^ Superior imaging modalities, such as molecular imaging using positron emission tomography (PET) and single-photon emission tomography (SPECT), with reliable detection of metastases, might crucially alter oncological outcomes for patients with PCa.^3^ The introduction of the prostate-specific membrane antigen (PSMA)–PET since 2011 has resulted in significant advances in the early detection of small lymph node and bone metastases compared with the conventional modalities.^2^ The main advantage of PSMA–PET is the simultaneous staging of both primary tumor, visceral, bone, and lymph node metastases.^4^ Food and Drug Administration (FDA)-approved radiotracers visualizing PSMA include [^68^Ga]­Ga-PSMA-11, [^18^F]­F-DCFPyL, and [^18^F]­F-PSMA-1007.^5^ Although the recent European Association of Urology 2021 guidelines on PCa do not yet recommend PSMA–PET as the upfront imaging modality for primary staging, its importance is recognized as the most accurate method for the detection of regional and distant metastases in the primary staging of PCa.^6^

The limited global availability of PET cameras brings challenges in the efficient implementation of PSMA imaging, even though PSMA–PET has proven its effectiveness in the detection of PCa. According to Eurostat, PET scanners were the least widely available imaging equipment in 2022, at most 0.49 units per 100,000 inhabitants. At the same time, the number of gamma cameras, including SPECT cameras, varies between 1.4 and 2.7 units per 100,000 inhabitants.^7^ The recent advancements in the development of new-generation SPECT cameras, the low cost of SPECT investigations, and the high prevalence of PCa, with the projected increase of diagnosed cases, support the development of PSMA imaging agents for SPECT imaging.^8^

Technetium-99m, as one of the most available SPECT radionuclides, may provide greater accessibility to PSMA-SPECT globally compared to ^68^Ga/^18^F-PSMA–PET.^9^ Technetium-99m with a half-life of 6 h and a γ-energy of 140.5 keV is widely utilized in nuclear medicine due to its photon energy convenient for SPECT, low dose burden to patients, low cost, and availability.^10^ The labeling chemistry of technetium-99m has been extensively studied and offers a wide range of methods, owing to the element’s numerous oxidation states.^11^ Over the past few years, various technetium-99m-labeled PSMA tracers have been developed, primarily based on low-molecular-weight PSMA inhibitors, and have been explored for imaging applications.^12,13^ Tracers based on the glutamate–urea–lysine (EuK) pharmacophore have demonstrated the greatest promise to date. Several small-molecule PSMA inhibitors for PCa SPECT imaging, such as [^99m^Tc]­Tc-PSMA-I&S, [^99m^Tc]­Tc-HYNIC-IPSMA, [^99m^Tc]­Tc-HYNIC-ALUG, [^99m^Tc]­Tc-PSMA-T4, and recently developed in our group, [^99m^Tc]­Tc-BQ0413, have progressed into clinical trials.^12–14^ [^99m^Tc]­Tc-BQ0413, containing a mercaptoacetyl-Glu-Glu-Glu (maE_3_) chelator, demonstrated low picomolar affinity to PSMA.^15^ The phase I clinical study confirmed the efficient tumor targeting, and high contrast visualization of PCa lesions was obtained 2 h after the administration of [^99m^Tc]­Tc-BQ0413. It was found that imaging contrast depends on the mass of injected tracer, and the optimal mass was 100 μg. The major clearance of the tracer was through glomerular filtration, and [^99m^Tc]­Tc-BQ0413 exhibited long activity retention in the kidneys.^14^ In this work, we build upon our previous developments by investigating how different mercaptoacetyl-based chelators influence pharmacokinetics and tumor targeting.

It is known that the labeling methods influence the overall biodistribution, excretion pathways, and washout rate due to different charge distribution, overall charge of the tracer, and residualizing properties of the label in cells after internalization.^16,17^ Therefore, the addition or removal of the charged molecular units as well as their positioning within the tracer have been extensively explored.^18^ The choice of chelating agent significantly impacts the stability, affinity, lipophilicity, and ultimately the pharmacokinetic behavior of the final technetium-99m-labeled radiotracers.^19^ The variation of amino acids in the peptide-based chelators for technetium-99m incorporation allows for a fine-tuning of the biodistribution properties of the labeled peptides.^20^ Our first attempt to decrease the retention of activity in normal organs, including PSMA-expressing organs as the kidneys, was to incorporate the mercaptoacetyl-Gly-Gly-Gly (maG_3_) chelator instead of maE_3_.^21^ This chelator is recognized for its mild residualizing properties.^22^ This led to the development of two new PSMA inhibitors, BQ0411 and BQ0412, that demonstrated decreased renal activity accumulation compared to the reference BQ0413. However, incorporation of the maG_3_ chelator resulted in an undesirable increase in the abdominal activity uptake, limiting the detection of metastases anatomically relevant to PCa.^21^ Our earlier studies revealed that a combination of serine and glutamic acid residues in the chelator leads to a relatively low renal activity uptake despite renal excretion of technetium-99m-labeled proteins, e.g., Affibody molecules.^23,24^ The maS_3_ chelator was used by Robu et al. in the development of the PSMA-I&S tracer for SPECT imaging for radioguided surgery.^25^ The tracer demonstrated favorable biodistribution for the next day imaging with high lesion-to-background contrast at the time of surgery and facilitated a cost-effective formulation of single-vial kits, streamlining clinical translation. However, the tracer demonstrated delayed whole-body and relatively late background clearance, particularly in the abdominal region, due to high lipophilicity.^25^

Taken together, with the above-mentioned findings, we hypothesized that incorporation of serine in the chelator might decrease activity retention in normal organs, including PSMA-expressing organs, while keeping the optimal hydrophilicity for renal clearance. In the present study, we designed and preclinically evaluated two new EuK-based PSMA-targeting tracers based on the structure of BQ0413: BQ0500 with a mercaptoacetyl-Glu-Ser-Glu chelator and BQ0501 with a mercaptoacetyl-Ser-Ser-Ser chelator (chemical structures of the studied tracers are presented in Figure 1 with defined overall charge after complexation, and the consensus structure of the Tc­(O)-complex^26^).

**Figure 1:**
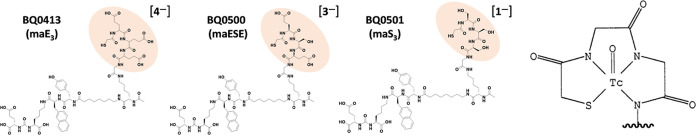
Chemical structures of BQ0413, BQ0500, and BQ0501.

## Materials and Methods

BQ0500 and BQ0501 (shown in Figure 1) were synthesized by Pepmic Co., Ltd. according to our molecular design. The mass spectrometry data for the synthesized tracers can be found in the Supporting Information (Figures S1–S3). PSMA-11 was purchased from ABX Advanced Biochemical Compounds (Radeberg, Germany). The PSMA-transfected PC3-pip cell line was issued by Prof. Martin G. Pomper (Johns Hopkins University, Baltimore, MD, USA). The prostate carcinoma cell line PC-3 was purchased from ATCC (Manassas, VA, USA). The cell lines were cultured in RPMI-1640 media, and PC3-pip cells were maintained with the addition of 10 mg/mL puromycin every second passage. Media supplements (fetal bovine serum, penicillin–streptomycin (100 IU/mL penicillin, 100 μg/mL streptomycin), 2 mM l-glutamine, and trypsin–ethylenediaminetetraacetic acid (EDTA) solution for cell detachment) were purchased from Avantor, VWR International (Sweden).

Technetium-99m was obtained as a pertechnetate by elution of an Ultra-TechneKow generator (Mallinckrodt, Petten, The Netherlands) with sterile 0.9% sodium chloride (Mallinckrodt, Petten, The Netherlands). Instant thin-layer chromatography (iTLC) analysis was performed by using iTLC strips (Varian, Lake Forest, CA, USA). iTLC was analyzed using a Cyclone Plus storage Phosphor System (PerkinElmer, Waltham, MA, USA). The radiochemical purity was quantified using high-performance liquid chromatography (HPLC). Radio-HPLC analysis was performed using a Hitachi Chromaster HPLC system with a radioactivity detector and Phenomenex Luna C18 column (100 Å; 150 × 4.6 mm^2^; 5 μm) at room temperature (20 °C). Solvent A was 0.1% trifluoroacetic acid (TFA) in H_2_O, solvent B was 0.1% TFA in acetonitrile (ACN), and the flow rate was 1 mL/min. For identity and purity analysis, a method with a gradient from 5 to 60% solvent B over 20 min was used.

Binding specificity and cellular processing experiments were performed using 35 × 10 mm^2^ Petri dishes (Corning Inc., Oneonta, N.Y., USA). Ligand Tracer experiments were performed in 100 × 20 mm^2^ Corning CellBIND surface dishes (Corning Inc., Oneonta, N.Y., USA) using LigandTracer Yellow Instruments (Ridgeview Instruments AB, Uppsala, Sweden). Radioactivity was measured using an automated gamma-spectrometer with a NaI (TI) detector (1480 Wizard, Wallac, Turku, Finland).

### Radiolabeling, Stability, and Distribution Coefficient (Log *D*)

BQ0413 was dissolved in phosphate-buffered saline (PBS) at a concentration of 0.7 mg/mL. BQ0500 was dissolved in PBS with the addition of 40% ethanol at a concentration of 1.1 mg/mL. BQ0501 was dissolved in water/ACN/NH_4_OH 5% (3:1:1) at a concentration of 1 mg/mL. The level of ACN and ethanol in the final solution was within the range approved by the FDA for residual solvents.^27^ The concentration of NH_4_OH was within the range approved by the guideline on water for pharmaceutical use.^28^ Single-step technetium-99m labeling was performed similarly to the previously described method.^15^ Briefly, 10 μg of the peptide was added to a freeze-dried kit (5 mg of gluconic acid sodium salt, 75 μg of stannous chloride, and 100 μg of EDTA), followed by freshly eluted [^99m^Tc]­Tc-pertechnetate (specific activity—20 MBq per 1 μg, total activity—180–200 MBq), and the vial was incubated for 60 min at 90 °C.

The radiochemical yield was analyzed using iTLC strips eluted with acetone (*R*_*f*_ = 0 for radiolabeled tracers, *R*_*f*_ = 1 for [^99m^Tc]­Tc-TcO_4_). The reduced hydrolyzed technetium colloid (RHT) in the mixture was determined using iTLC, eluted with a pyridine/acetic acid/water (10:6:3) mobile phase (*R*_*f*_ = 1 for [^99m^Tc]­Tc-colloid, *R*_*f*_ = 0 for other forms of technetium-99m and radiolabeled tracers).

To evaluate the label’s stability, 10 μL (0.25 μg) of radiolabeled conjugates was incubated with 300× molar excess of cysteine or with PBS for 1 h at room temperature. The test was run in triplicate. The release of free technetium-99m was controlled by using iTLC and radio-HPLC analysis. The stability in serum was analyzed by mixing the radioconjugates (0.25 μg in 10 μL) with murine serum or PBS (40 μL) as a control. The samples were incubated for 1 h at 37 °C and analyzed by iTLC. The experiment was performed in triplicate. The octanol–water distribution coefficient (Log *D*) was determined experimentally by the addition of 100 pmol of the radiolabeled tracer to an Eppendorf tube containing PBS (500 μL) and *n*-octanol (500 μL), pH ∼ 7.4. The solution was vortexed and centrifuged, and aliquots (50 μL) from each phase were collected and measured using a gamma counter. The experiment was performed in triplicate.

### In Vitro Assays

For the in vitro binding specificity test, approximately 8 × 10^5^ PC3-pip cells per well were seeded 24 h before the experiment. On the day of the experiment, cells in three control dishes were presaturated with a 250-fold excess of nonlabeled PSMA-11 (*K*_D_ = 0.5 ± 0.2 nM)^29^ for 15 min, while the second set was treated with complete media. Technetium-99m-labeled tracers were added to each well to reach a concentration of 2 nM. After 1 h of incubation at 37 °C, cells were washed with PBS solution, detached, and collected. Cell-associated activity was measured using a gamma counter and presented as the percentage of added activity.

The binding kinetics of technetium-99m-labeled BQ0413, BQ0500, and BQ0501 were determined in PC3-pip cells in real-time using LigandTracer Yellow (Ridgeview Instruments AB, Uppsala, Sweden). The activity binding was recorded with 5 nM radiolabeled tracers for 150 min. After the binding kinetics were measured, the medium containing the labeled tracer was replaced with fresh medium, and the dissociation was monitored over 7 h. The association rate (*k*_a_) and the dissociation rate (*k*_d_) were computed using a 1:2 kinetic binding model in TraceDrawer software (Ridgeview Instruments AB, Uppsala, Sweden), and the equilibrium dissociation constant *K*_D_ was calculated.

To evaluate cellular processing of technetium-99m-labeled BQ0500 and BQ0501, PC3-pip cells were incubated with the radiolabeled tracer (2 nM), and at 1, 2, 4, and 8 h of continuous incubation, the membrane-bound and internalized fractions were collected as previously described using 4 M urea solution in 0.2 M glycine buffer (pH 2) to collect membrane-bound fraction and 1 N solution of NaOH to collect the internalized fraction.^15^ The activity in the samples was measured, and the percentage of membrane-bound and internalized activity was calculated. The experiments were performed in triplicate.

### In Vivo Assays

Mice for the in vivo experiments were purchased from Scanbur A/S (Sollentuna, Sweden). The in vivo studies were conducted in accordance with the guidelines of the Declaration of Helsinki and were approved by the Ethics Committee for Animal Research in Uppsala, Sweden (approval number: 5.8.18-00473/2021, dated February 26, 2021).

The injected masses for the in vivo experiments were chosen based on the findings for [^99m^Tc]­Tc-BQ0413: to study the general biodistribution of radiolabeled tracers in normal mice (NMRI), 40 pmol of tracer was used to avoid blocking of PSMA-mediated uptake in kidneys and salivary glands, to study tumor targeting, 5 nmol of tracer was used to decrease uptake in PSMA-expressing organs.^15^

For NMRI mice, each mouse was intravenously injected with 40 pmol of a radiotracer (60 kBq; the mass of the injected compound was adjusted with unlabeled tracer). The mice were euthanized after lethal intraperitoneal injection of ketamine/xylazine, followed by exsanguination 3 and 24 h pi. The organs of interest were collected, weighed, and measured for their activity content by using the gamma counter. The activity uptake in organs was calculated as the percentage of injected activity per gram (%IA/g) for organs and tissues, and as %IA for the remaining carcass and the rest of the intestines with content.

PSMA-expressing PCa xenografts were implanted in BALB/c nu/nu mice by subcutaneous injection of the PC3-pip cell suspension in PBS (10^7^ cells/animal in 100 μL). The average animal weight in the experiment was 18 ± 2 g. The average tumor weight was 0.4 ± 0.2 g. For in vivo specificity control, 10^7^ cells/animal PSMA-negative PC-3 cells were implanted. The average animal weight was 18 ± 1 g. The average tumor weight was 0.1 ± 0.1 g. Four mice per data point were used in the in vivo experiments.

For tumor-bearing mice, each mouse was intravenously injected with 5 nmol of [^99m^Tc]­Tc-BQ0413, [^99m^Tc]­Tc-BQ0500, and [^99m^Tc]­Tc-BQ0501 (60 kBq; the mass of the injected compound was adjusted with an unlabeled tracer). The mice were euthanized after lethal intraperitoneal injection of ketamine/xylazine, followed by exsanguination 1 and 3 h pi, similarly to the procedure described above.

The in vivo specificity for [^99m^Tc]­Tc-BQ0500 and [^99m^Tc]­Tc-BQ0501 was investigated by using PSMA-negative PC-3 (prostate carcinoma) xenografts. Each mouse was intravenously injected with 5 nmol of the tested tracer (60 kBq, the mass of the injected tracer was adjusted with an unlabeled compound). Animals were euthanized 3 h pi, organs were collected, and uptake activity was measured according to the protocol described above.

For SPECT/CT imaging, the mice were intravenously injected with 5 nmol (1 MBq) of [^99m^Tc]­Tc-BQ0413, [^99m^Tc]­Tc-BQ0500, and [^99m^Tc]­Tc-BQ0501. Whole-body SPECT/CT scans were performed for 3 h using nanoScan SPECT/CT (Mediso Medical Imaging Systems Ltd., Budapest, Hungary). SPECT raw data were reconstructed using Tera-Tomo 3D SPECT reconstruction technology (version 3.00.020.000; Mediso Medical Imaging Systems Ltd., Budapest, Hungary): normal dynamic range; 30 iterations; and one subset. CT data were reconstructed using Filter Back Projection in Nucline 2.03 Software (Mediso Medical Imaging Systems Ltd., Budapest, Hungary). SPECT and CT files were fused using Nucline 2.03 Software and are presented as maximum intensity projections on the RGB color scale.

### Data Analysis

The obtained values are presented as the average with a standard deviation (SD). Data were assessed either by an unpaired, two-tailed *t*-test or by one-way analysis of variance (ANOVA) with Bonferroni correction for multiple comparisons using GraphPad Prism software version 10.00 for Windows, GraphPad Software, San Diego, California. The difference was considered significant when the *p* value was less than 0.05.

## Results

### Radiolabeling and In Vitro Characterization

The reference BQ0413 was labeled with a radiochemical yield of 98.5 ± 0.5%, *n* = 5, as determined by iTLC. BQ0500 and BQ0501 were labeled with radiochemical yields over 97% (RCY for [^99m^Tc]­Tc-BQ0500 was 98 ± 0.6% (*n* = 5), RCY for [^99m^Tc]­Tc-BQ0501 was 97 ± 0.4% (*n* = 5)). RHT was less than 3% for all three tracers. To cross-validate radio-iTLC data, radio-HPLC analysis was performed. UV spectra of nonlabeled BQ0500 and BQ0501, along with the corresponding radio-HPLC chromatograms of [^99m^Tc]­Tc-BQ0500 and [^99m^Tc]­Tc-BQ0501, are presented in Figure 2. The results confirmed the findings from the iTLC analysis. No further purification was performed prior to the following in vitro and in vivo studies.

**Figure 2:**
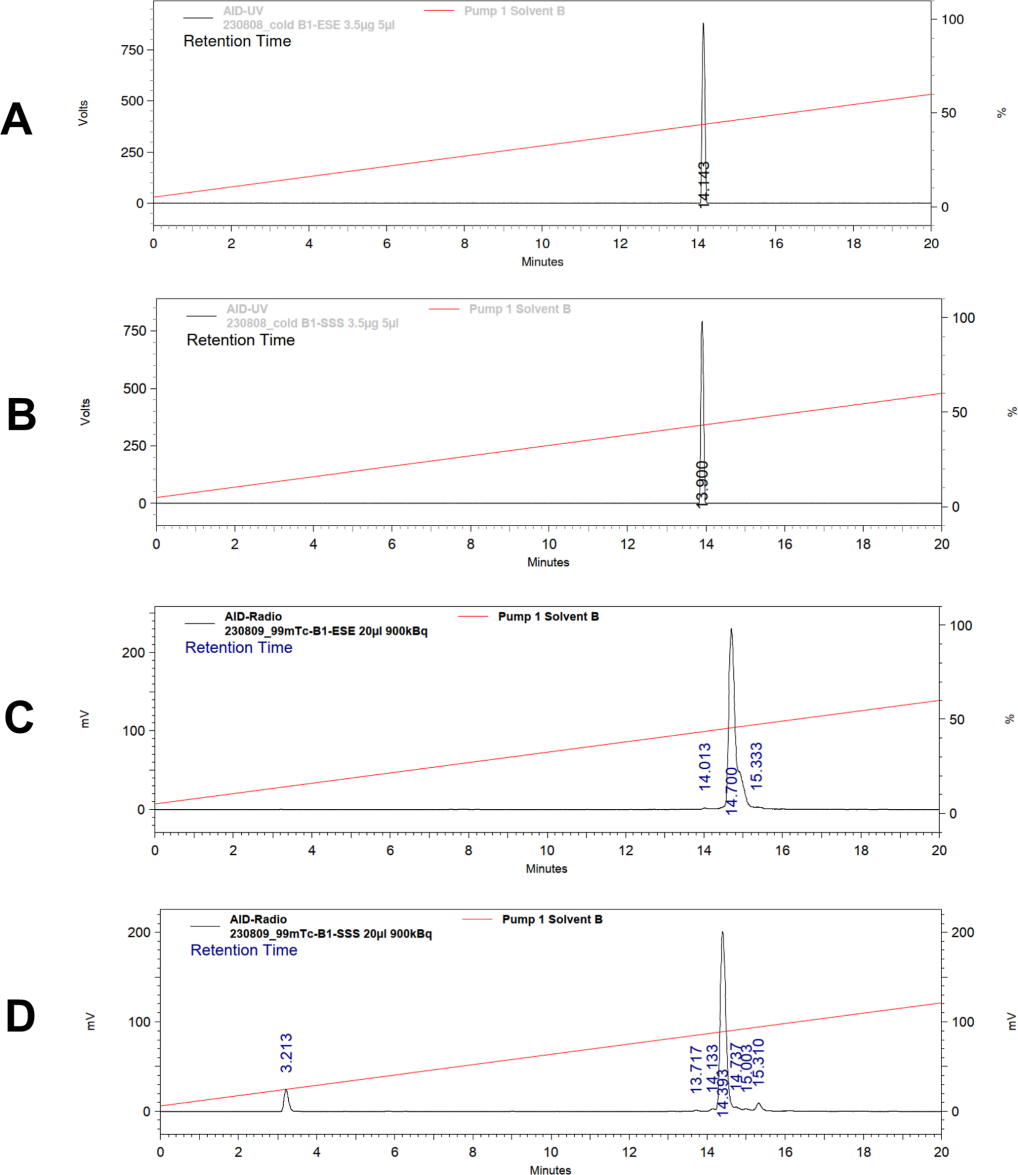
Reversed-phase HPLC chromatograms of nonlabeled BQ0500 (A) and BQ0501 (B) and radiochromatograms of [^99m^Tc]­Tc-BQ0500 (C) and [^99m^Tc]­Tc-BQ0501 (D). The retention times are expressed in minutes.

Both [^99m^Tc]­Tc-BQ0500 and [^99m^Tc]­Tc-BQ0501 were stable in vitro during 1 h of incubation with a 300-fold molar excess of cysteine or in PBS at room temperature (Figures S4A,B and S5A,B). After 1 h of incubation in murine serum and PBS, as a control, at 37 °C, both radiotracers demonstrated high stability, with less than 3% release of free technetium-99m. [^99m^Tc]­Tc-BQ0500 retained 99.7 ± 0.3% (*n* = 3) of intact tracer in murine serum and 99.0 ± 0.5% (*n* = 3) in PBS. [^99m^Tc]­Tc-BQ0501 showed 98.7 ± 0.6% (*n* = 3) of intact tracer in murine serum and 97.4 ± 0.9% (*n* = 3) in PBS. The octanol–water distribution coefficient showed similar Log *D* values for all three tracers (−2.4 ± 0.1 for [^99m^Tc]­Tc-BQ0501 and −2.5 ± 0.1 for [^99m^Tc]­Tc-BQ0500 and [^99m^Tc]­Tc-BQ0413).

The results of the in vitro binding specificity test are listed in Figure 3. The high activity uptake by PC3-pip cells (71 ± 4% of added activity for [^99m^Tc]­Tc-BQ0500 and 62 ± 7% for [^99m^Tc]­Tc-BQ0501) was significantly (*p* < 0.0001) decreased when the cells were pretreated with an excess amount of the nonlabeled PSMA-inhibitor PSMA-11.

**Figure 3:**
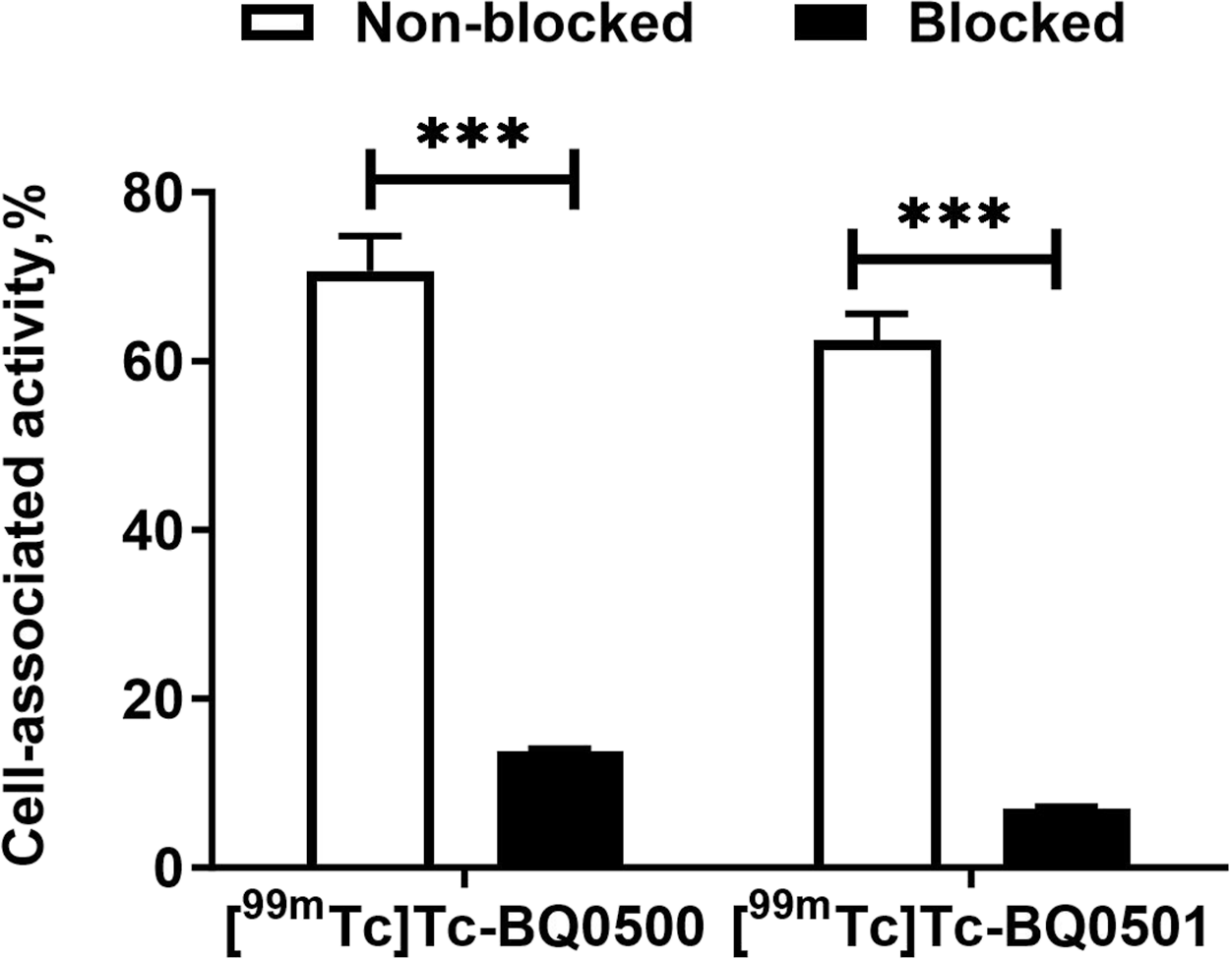
In vitro binding specificity of [^99m^Tc]­Tc-BQ0500 and [^99m^Tc]­Tc-BQ0501 on the PC3-pip cell line. For presaturation of PSMA, a 250-fold molar excess of nonlabeled PSMA-11 was added before adding labeled tracers. The error bars represent the SD. *** indicates a *p*-value less than 0.0001 in an unpaired *t*-test.

The cellular processing of [^99m^Tc]­Tc-BQ0500 and [^99m^Tc]­Tc-BQ0501 by PC3-pip cells (Figure 4) demonstrated an increase in cell-associated activity up to 4 h of continuous incubation. Internalization was relatively rapid, reaching the maximum of internalized activity after 8 h (42–43% of cell-associated activity).

**Figure 4:**
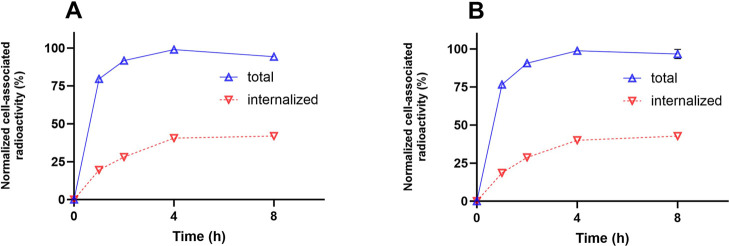
Cellular processing of [^99m^Tc]­Tc-BQ0500 (A) and [^99m^Tc]­Tc-BQ0501 (B) by PC3-pip cells during continuous incubation. Cells were incubated with 2 nM of each peptide at 37 °C. Data are presented as the mean of three samples ± SD. Error bars might not be seen when they are smaller than data point symbols.

The binding kinetics of [^99m^Tc]­Tc-BQ0500 and [^99m^Tc]­Tc-BQ0501 to PSMA were measured in real time using living PC3-pip cells and compared with [^99m^Tc]­Tc-BQ0413 (Figure 5). The interaction model 1:2 provided the best fitting for [^99m^Tc]­Tc-BQ0500 and [^99m^Tc]­Tc-BQ0501. Rapid on-rate and slow off-rate were observed for the new radiotracers (Table 1). For [^99m^Tc]­Tc-BQ0500, both *K*_D1_ and *K*_D2_ were within the picomolar range. For [^99m^Tc]­Tc-BQ0501, *K*_D1_ within the picomolar range was the predominant interaction, and *K*_D2_ was within the nanomolar range. For [^99m^Tc]­Tc-BQ0413, the 1:1 interaction model showed the best fitting with a *K*_D_ value within a low picomolar range (89 × 10^–12^). For all three tested constructs, the most abundant interaction, *B*_max_ (binding maximum), was in the picomolar range according to the obtained values (Table 1).

**Figure 5:**
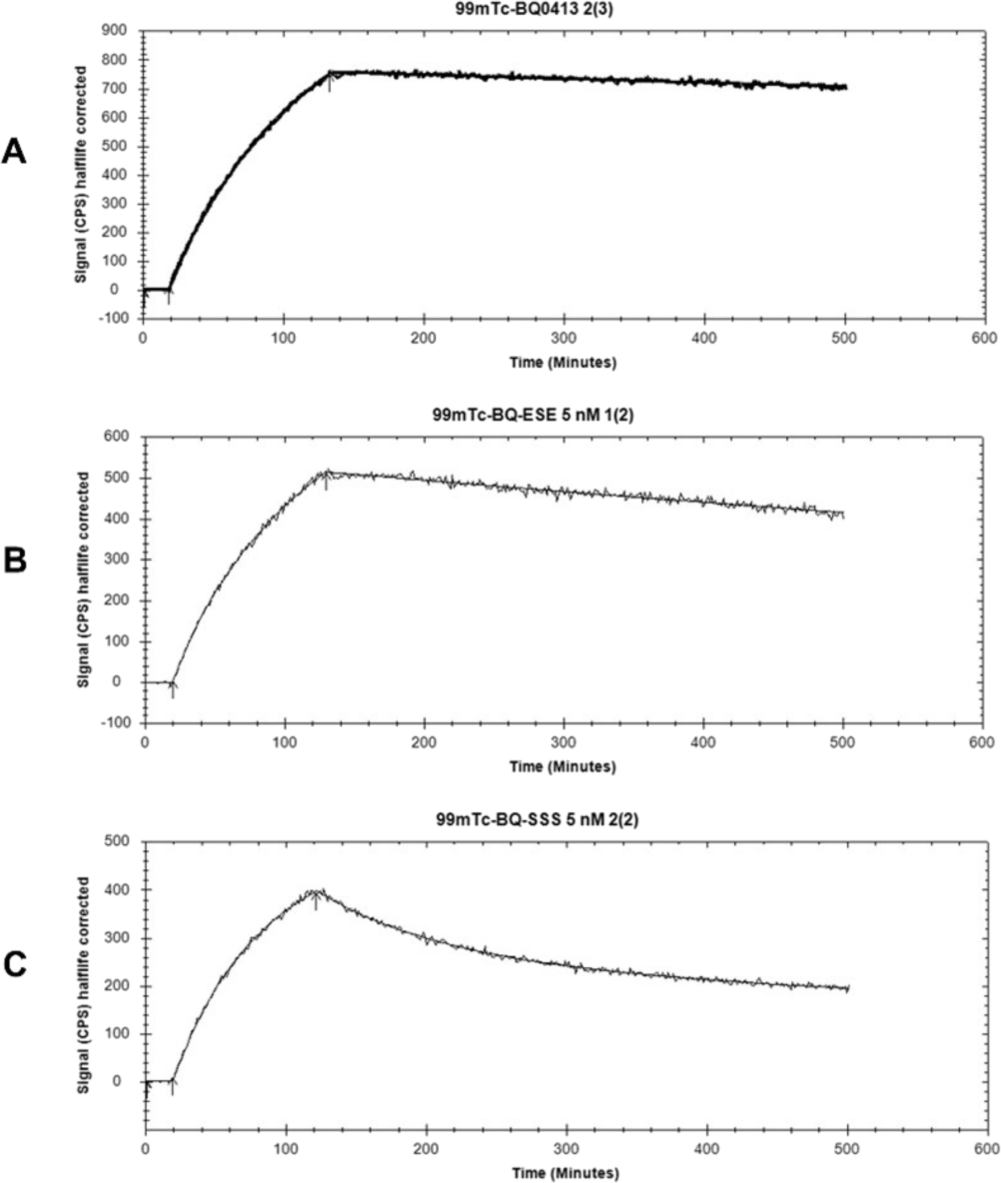
Sensorgrams obtained using LigandTracer Yellow for [^99m^Tc]­Tc-BQ0413 (A), [^99m^Tc]­Tc-BQ0500 (B), and [^99m^Tc]­Tc-BQ0501 (C). The concentration during association measurements was 5 nM for each radiolabeled peptide.

**Table 1:** Affinity Measurements of [^99m^Tc]­Tc-BQ0500, [^99m^Tc]­Tc-BQ0501, and [^99m^Tc]­Tc-BQ0413^a^

interaction constants	[^99m^Tc]Tc-BQ0500	[^99m^Tc]Tc-BQ0501	[^99m^Tc]Tc-BQ0413
*k*_a1_ (M^–1^ s^–1^)	8.8 × 10^4^	2.7 × 10^4^	3.3 × 10^4^
*k*_d1_ (s^–1^)	9.0 × 10^–6^	1.2 × 10^–5^	2.9 × 10^–6^
*K*_D1_ (M)	295 × 10^–12^	474 × 10^–12^	89 × 10^–12^
*B*_max1_ (CPS)	881	492	947
*k*_a2_ (M^–1^ s^–1^)	3.0 × 10^4^	4.9 × 10^4^	
*k*_d2_ (s^–1^)	1.6 × 10^–5^	1.6 × 10^–4^	
*K*_D2_ (M)	52 × 10^–12^	3.6 × 10^–9^	
*B*_max2_ (CPS)	40	272	

a *k*_a_—association constant, *k*_d_—dissociation constant, *K*_D_—equilibrium dissociation constant, *B*_max_—binding maximum.

The biodistribution properties of [^99m^Tc]­Tc-BQ0500, [^99m^Tc]­Tc-BQ0501, and [^99m^Tc]­Tc-BQ0413 were compared at 3 and 24 h in NMRI mice after the injection of 40 pmol of tracer per animal (Table 2). All three tested constructs demonstrated fast blood clearance (activity concentration in blood was <1% %IA/g at 3 h pi) and were predominantly excreted via the kidneys. At 3 h pi, [^99m^Tc]­Tc-BQ0500 and [^99m^Tc]­Tc-BQ0501 had significantly lower activity uptake in blood, liver, and stomach compared to that of the reference [^99m^Tc]­Tc-BQ0413. [^99m^Tc]­Tc-BQ0501 had significantly lower activity uptake in kidneys, salivary gland, lungs, spleen, and carcass compared to [^99m^Tc]­Tc-BQ0500 and reference [^99m^Tc]­Tc-BQ0413. At the same time, [^99m^Tc]­Tc-BQ0501 demonstrated the highest activity uptake in intestines with content (2.6-fold higher compared to reference [^99m^Tc]­Tc-BQ0413 and 2-fold higher compared to [^99m^Tc]­Tc-BQ0500). At 24 h pi, the activity uptake in all organs significantly decreased compared to 3 h pi for all three tested tracers. [^99m^Tc]­Tc-BQ0501 demonstrated significantly lower activity uptake in kidneys, salivary gland, lungs, and carcass compared to [^99m^Tc]­Tc-BQ0500 and reference [^99m^Tc]­Tc-BQ0413. Markedly, [^99m^Tc]­Tc-BQ0501 showed the lowest activity uptake in intestines with content (4.2-fold lower compared to that of reference [^99m^Tc]­Tc-BQ0413). [^99m^Tc]­Tc-BQ0500 and [^99m^Tc]­Tc-BQ0501 had significantly lower activity uptake in the blood, liver, and stomach compared to [^99m^Tc]­Tc-BQ0413. [^99m^Tc]­Tc-BQ0501 demonstrated significantly lower activity uptake in the spleen compared to that of the reference [^99m^Tc]­Tc-BQ0413.

**Table 2:** In Vivo Biodistribution of [^99m^Tc]­Tc-BQ0500, [^99m^Tc]­Tc-BQ0501, and [^99m^Tc]­Tc-BQ0413 in NMRI Mice at 3 and 24 h after Injection of 40 pmol/Mouse^a^

	[^99m^Tc]Tc-BQ0500		[^99m^Tc]Tc-BQ0501		[^99m^Tc]Tc-BQ0413	
Organ	3 h pi	24 h pi	3 h pi	24 h pi	3 h pi	24 h pi
blood	0.31 ± 0.06^b^ ^,^ ^d^	0.049 ± 0.009^c^	0.29 ± 0.04^b^ ^,^ ^e^	0.008 ± 0.001^e^	0.5 ± 0.1^b^	0.05 ± 0.01
salivary	1.10 ± 0.19^b^ ^,^ ^c^ ^,^ ^d^	0.09 ± 0.02^c^ ^d^	0.25 ± 0.04^b^ ^e^	0.011 ± 0.002^e^	3.0 ± 0.1^b^	0.30 ± 0.01
lung	0.9 ± 0.1^b^ ^,^ ^c^	0.06 ± 0.02^c^ ^d^	0.29 ± 0.04^b^ ^e^	0.012 ± 0.003^e^	1.0 ± 0.3^b^	0.18 ± 0.03
liver	0.30 ± 0.08^b^ ^d^	0.036 ± 0.009^d^	0.43 ± 0.04^b^ ^e^	0.014 ± 0.002^e^	0.7 ± 0.1^b^	0.21 ± 0.03
spleen	1.3 ± 0.4^b^ ^c^	0.07 ± 0.01	0.25 ± 0.03^b^ ^e^	0.013 ± 0.007^e^	1.2 ± 0.3^b^	0.3 ± 0.2
stomach	0.32 ± 0.07^b^ ^d^	0.05 ± 0.03^d^	0.12 ± 0.02^b^ ^e^	0.02 ± 0.01^e^	3.1 ± 0.5^b^	1.0 ± 0.5
kidney	250 ± 43^b^ ^c^	36 ± 5^c^ ^d^	149 ± 26^b^ ^e^	2.1 ± 0.2^e^	242 ± 43^b^	56 ± 14
intestines with content	5.1 ± 0.4^b^ ^c^	0.6 ± 0.2^c^	10.0 ± 0.4^b^ ^e^	0.12 ± 0.02^e^	3.8 ± 0.4^b^	0.5 ± 0.2
carcass	9 ± 3^b^ ^c^	0.7 ± 0.3^c^ ^d^	2.8 ± 0.6^b^ ^e^	0.11 ± 0.01^e^	9 ± 1^b^	1.4 ± 0.2

a Data are expressed as the percentage of administered activity (injected probe) per gram of tissue (%IA/g). Activity in intestines with content and carcass is presented as %IA/sample. The data are presented as the average (n = 4) and SD.

b Significant difference (*p* < 0.05 in 2-tailed *t* test) between 3 h pi and 24 h pi for the corresponding tracer.

c significant difference (*p* < 0.05) between [^99m^Tc]­Tc-BQ0500 and [^99m^Tc]­Tc-BQ0501.

d significant difference (*p* < 0.05) between [^99m^Tc]­Tc-BQ0500 and [^99m^Tc]­Tc-BQ0413.

e significant difference (*p* < 0.05) between [^99m^Tc]­Tc-BQ0501 and [^99m^Tc]­Tc-BQ0413. ANOVA test (Bonferroni’s multiple comparisons test) was performed to test for a significant (*p* < 0.05) difference.

The tumor-targeting properties of [^99m^Tc]­Tc-BQ0500, [^99m^Tc]­Tc-BQ0501, and [^99m^Tc]­Tc-BQ0413 were investigated in PC3-pip tumor-bearing mice 1 and 3 h after injection of 5 nmol/animal (Table 3). At 1 h pi, there was no significant difference in tumor activity uptake for [^99m^Tc]­Tc-BQ0500 and [^99m^Tc]­Tc-BQ0501. At 3 h pi, new tracers had about 2-fold higher activity uptake in tumors compared to [^99m^Tc]­Tc-BQ0413. The overall clearance for all constructs was rapid (<2% %IA/g in blood at 1 h pi), and excretion was predominantly via the kidneys. [^99m^Tc]­Tc-BQ0501 at 1 h pi showed activity uptake in kidneys similar to [^99m^Tc]­Tc-BQ0413 and 2.4-fold lower compared to [^99m^Tc]­Tc-BQ0500. At 3 h pi, the activity uptake in the kidneys for [^99m^Tc]­Tc-BQ0501 was 8.3-fold lower compared to that for [^99m^Tc]­Tc-BQ0500 and 2.3-fold lower compared to that for [^99m^Tc]­Tc-BQ0413. At the same time, [^99m^Tc]­Tc-BQ0501 had the highest activity uptake in the liver 1 h pi and intestines with content at both time points. [^99m^Tc]­Tc-BQ0500 also had higher activity uptake in intestines with content when compared to that of reference [^99m^Tc]­Tc-BQ0413, 5.3-fold, but to a lesser extent. [^99m^Tc]­Tc-BQ0413 had significantly higher activity uptake in the salivary gland compared to that of [^99m^Tc]­Tc-BQ0500 and [^99m^Tc]­Tc-BQ0501 at both time points. At 3 h pi, [^99m^Tc]­Tc-BQ0500 had significantly higher activity uptake in the lungs, liver, spleen, and stomach compared to [^99m^Tc]­Tc-BQ0501. Both new tracers demonstrated 2-fold lower activity uptake in bones compared to [^99m^Tc]­Tc-BQ0413.

**Table 3:** In Vivo Biodistribution of [^99m^Tc]­Tc-BQ0500, [^99m^Tc]­Tc-BQ0501, and [^99m^Tc]­Tc-BQ0413 in PC3-pip Tumor-Bearing Mice at 1 and 3 h after Injection of 5 nmol/Mouse^a^

	[^99m^Tc]Tc-BQ0500		[^99m^Tc]Tc-BQ0501		[^99m^Tc]Tc-BQ0413	
organ	1 h pi	3 h pi	1 h pi	3 h pi	1 h pi	3 h pi
blood	1.1 ± 0.2^b^	0.3 ± 0.1^c^ ^,^ ^d^	1.6 ± 0.5^b^	0.14 ± 0.07	1.0 ± 0.3^b^	0.17 ± 0.04
salivary	0.7 ± 0.1^b^ ^,^ ^d^	0.2 ± 0.1^d^	0.6 ± 0.2^b^ ^,^ ^e^	0.14 ± 0.06^e^	1.4 ± 0.2^b^	0.5 ± 0.1
lung	1.3 ± 0.2^b^	0.4 ± 0.1^c^	1.6 ± 0.4^b^	0.21 ± 0.07	1.2 ± 0.2^b^	0.3 ± 0.1
liver	0.9 ± 0.2^b^	0.4 ± 0.1^c^	1.8 ± 0.7^b^	0.18 ± 0.06	1.3 ± 0.4^b^	0.3 ± 0.1
spleen	2.5 ± 0.2^b^	1.4 ± 0.3^c^	2.4 ± 0.5^b^	0.3 ± 0.2	1.9 ± 0.8	0.9 ± 0.5
pancreas	0.4 ± 0.1^b^	0.3 ± 0.2	0.45 ± 0.05^b^	0.1 ± 0.1	0.6 ± 0.1^b^	0.13 ± 0.02
stomach	0.47 ± 0.02^d^	1.6 ± 1.2^c^	0.6 ± 0.2^b^ ^e^	0.14 ± 0.06	1.5 ± 0.4	0.6 ± 0.2
small intestines	0.6 ± 0.2^c^	0.4 ± 0.2	1.4 ± 0.6^b^ ^e^	0.17 ± 0.03	0.5 ± 0.1^b^	0.2 ± 0.1
kidney	51 ± 3^b^ ^c^	33 ± 3^c^,^d^	21 ± 6^b^	4 ± 2^e^	23 ± 3^b^	9 ± 2
tumor	29 ± 3	31 ± 4^d^	27 ± 7	35 ± 4^e^	19 ± 4	17 ± 3
muscle	0.3 ± 0.1^b^	0.10 ± 0.03	0.3 ± 0.1^b^	0.08 ± 0.04	0.3 ± 0.1^b^	0.06 ± 0.02
bone	0.5 ± 0.1^b^,^d^	0.10 ± 0.02	0.4 ± 0.1^b^ ^e^	0.07 ± 0.03^e^	0.9 ± 0.3^b^	0.2 ± 0.1
intestines with content	3.9 ± 0.4^b^,^c^,^d^	5.4 ± 0.3^c^,^d^	9 ± 1^b^ ^e^	11.0 ± 0.5^e^	1.7 ± 0.4	1.7 ± 0.2
carcass	7 ± 1^b^	4 ± 1^d^	8 ± 2^b^	3 ± 1	8 ± 2^b^	1.7 ± 0.3

a Data are expressed as the percentage of administered activity (injected probe) per gram of tissue (%IA/g). Activity in intestines with content and carcass is presented as %IA/sample. The data are presented as the average (*n* = 4) and SD.

b Significant difference (*p* < 0.05 in two-tailed *t*-test) between 1 h pi and 3 h pi for the corresponding tracer.

c Significant difference (*p* < 0.05) between [^99m^Tc]­Tc-BQ0500 and [^99m^Tc]­Tc-BQ0501.

d Significant difference (*p* < 0.05) between [^99m^Tc]­Tc-BQ0500 and [^99m^Tc]­Tc-BQ0413.

e Significant difference (*p* < 0.05) between [^99m^Tc]­Tc-BQ0501 and [^99m^Tc]­Tc-BQ0413. ANOVA test (Bonferroni’s multiple comparisons test) was performed to test for a significant (*p* < 0.05) difference.

Tumor-to-organ ratios (T/O) were the highest at 3 h pi when the activity was eliminated from the blood pool (Table 4). The T/O ratios increased from 1 to 3 h pi for all tested tracers in all organs, except the tumor-to-pancreas, tumor-to-stomach, and tumor-to-small intestines for [^99m^Tc]­Tc-BQ0500. [^99m^Tc]­Tc-BQ0501 had significantly higher tumor to anatomically relevant organ ratios (T/lung, T/liver, T/bone) among the tested tracers.

**Table 4:** Tumor-to-Organ Ratios of [^99m^Tc]­Tc-BQ0500, [^99m^Tc]­Tc-BQ0501, and [^99m^Tc]­Tc-BQ0413 in PC3-pip Tumor-Bearing Mice at 1 and 3 h after Injection of 5 nmol/Mouse^a^

	[^99m^Tc]Tc-BQ0500		[^99m^Tc]Tc-BQ0501		[^99m^Tc]Tc-BQ0413	
organ	1 h pi	3 h pi	1 h pi	3 h pi	1 h pi	3 h pi
blood	28 ± 6^b^	109 ± 21	19 ± 8^b^	299 ± 160^e^	22 ± 12^b^	94 ± 3
salivary	42 ± 8^b^ ^,^ ^d^	141 ± 42	47 ± 19^b^ ^,^ ^e^	302 ± 146^e^	14 ± 3^b^	31 ± 3
lung	23 ± 4^b^	71 ± 15^c^	17 ± 6^b^	178 ± 61^e^	16 ± 5^b^	52 ± 8
liver	32 ± 7^b^	79 ± 22^c^	17 ± 7^b^	222 ± 89^e^	17 ± 9^b^	48 ± 5
spleen	12 ± 2^b^	22 ± 5^c^	12 ± 5^b^	121 ± 47^e^	9 ± 3^b^	16 ± 4
pancreas	69 ± 13	140 ± 82^c^	62 ± 24^b^	463 ± 135^e^	38 ± 5^b^	135 ± 11
stomach	61 ± 7^d^	38 ± 39^c^	46 ± 18^b^ ^,^ ^e^	287 ± 102^e^	13 ± 2^b^	24 ± 2
small intestines	51 ± 20	94 ± 38^c^	22 ± 11^b^	209 ± 46	41 ± 17^b^	126 ± 57
kidney	0.6 ± 0.1^b^ ^,^ ^c^	1.0 ± 0.2^c^	1.3 ± 0.5^b^	10 ± 2^e^	0.9 ± 0.3^b^	1.9 ± 0.3
muscle	101 ± 28^b^	332 ± 99	86 ± 34^b^	504 ± 195	62 ± 25^b^	284 ± 19
bone	65 ± 15^b^ ^,^ ^d^	341 ± 100^d^	62 ± 18^b^ ^,^ ^e^	515 ± 113^e^	27 ± 17^b^	114 ± 29

a The data are presented as the average (*n* = 4) and SD.

b Significant difference (*p* < 0.05 in two-tailed *t*-test) between 1 h pi and 3 h pi for the corresponding tracer.

c Significant difference (*p* < 0.05) between [^99m^Tc]­Tc-BQ0500 and [^99m^Tc]­Tc-BQ0501.

d Significant difference (*p* < 0.05) between [^99m^Tc]­Tc-BQ0500 and [^99m^Tc]­Tc-BQ0413.

e Significant difference (*p* < 0.05) between [^99m^Tc]­Tc-BQ0501 and [^99m^Tc]­Tc-BQ0413. ANOVA test (Bonferroni’s multiple comparisons test) was performed to test for a significant (*p* < 0.05) difference.

[^99m^Tc]­Tc-BQ0500 and [^99m^Tc]­Tc-BQ0501 were then tested for PSMA-targeting specificity in vivo. Both constructs had significantly higher (*p* < 0.05, unpaired *t*-test) uptake in PSMA-positive PC3-pip than in PSMA-negative PC-3 xenografts (Figure 6 and Table S1).

**Figure 6:**
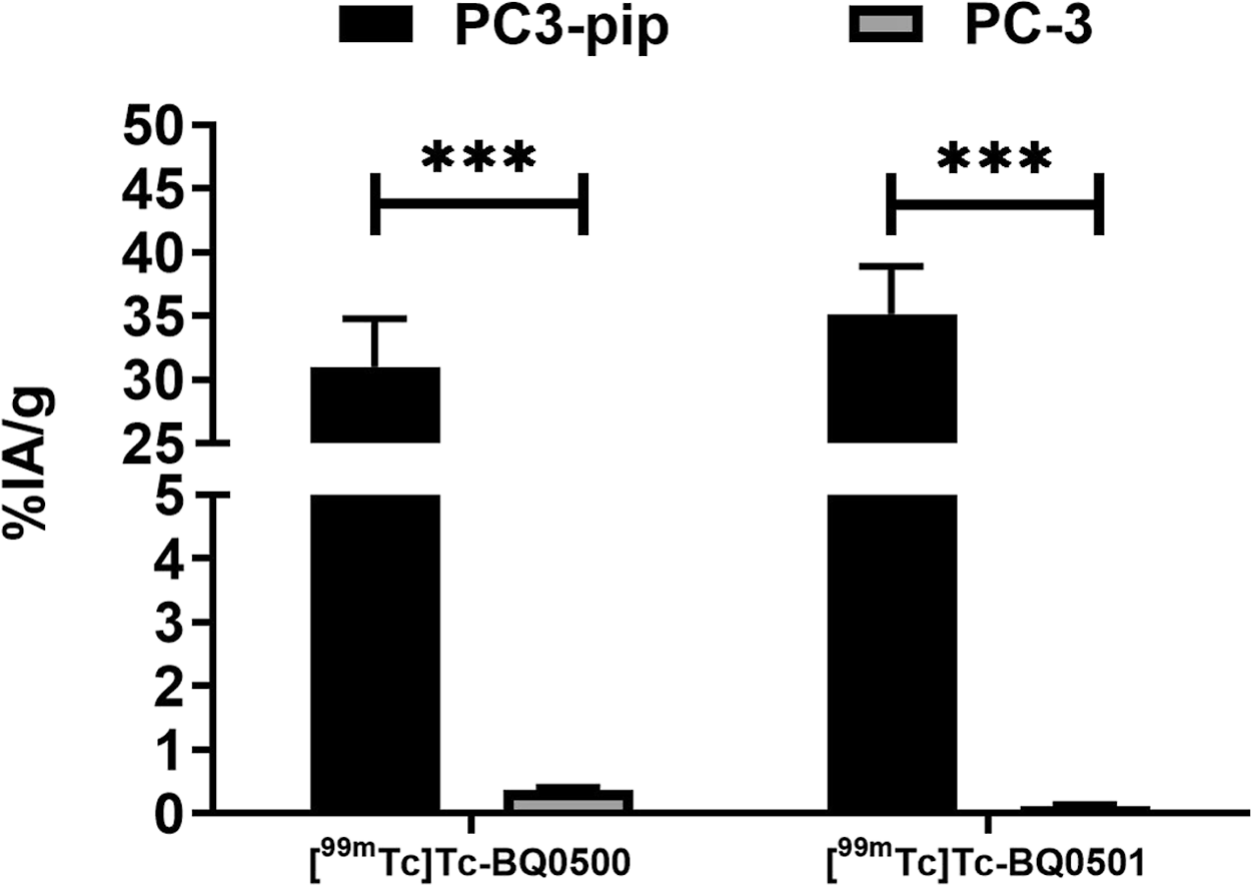
Comparison of tumor uptake of [^99m^Tc]­Tc-BQ0500 and [^99m^Tc]­Tc-BQ0501 in xenografts with high PSMA expression (PC3-pip) and low PSMA expression (PC-3) in BALB/c nu/nu mice. Data are presented as mean values and SDs for four mice. *** indicates a *p*-value less than 0.05 in a two-tailed *t*-test.

NanoScan SPECT/CT images of mice injected with 5 nmol of [^99m^Tc]­Tc-BQ0413, [^99m^Tc]­Tc-BQ0500, and [^99m^Tc]­Tc-BQ0501 (Figure 7) confirmed the findings from ex vivo biodistribution measurements. PSMA-expressing tumors were clearly visualized for all three conjugates at 3 h pi. Kidney uptake and renal excretion were observed for [^99m^Tc]­Tc-BQ0413 and [^99m^Tc]­Tc-BQ0500. For [^99m^Tc]­Tc-BQ0501, besides accumulation in the tumor, activity in the gastrointestinal tract was visualized. Uptake in other tissues was much lower compared to the tumor uptake.

**Figure 7:**
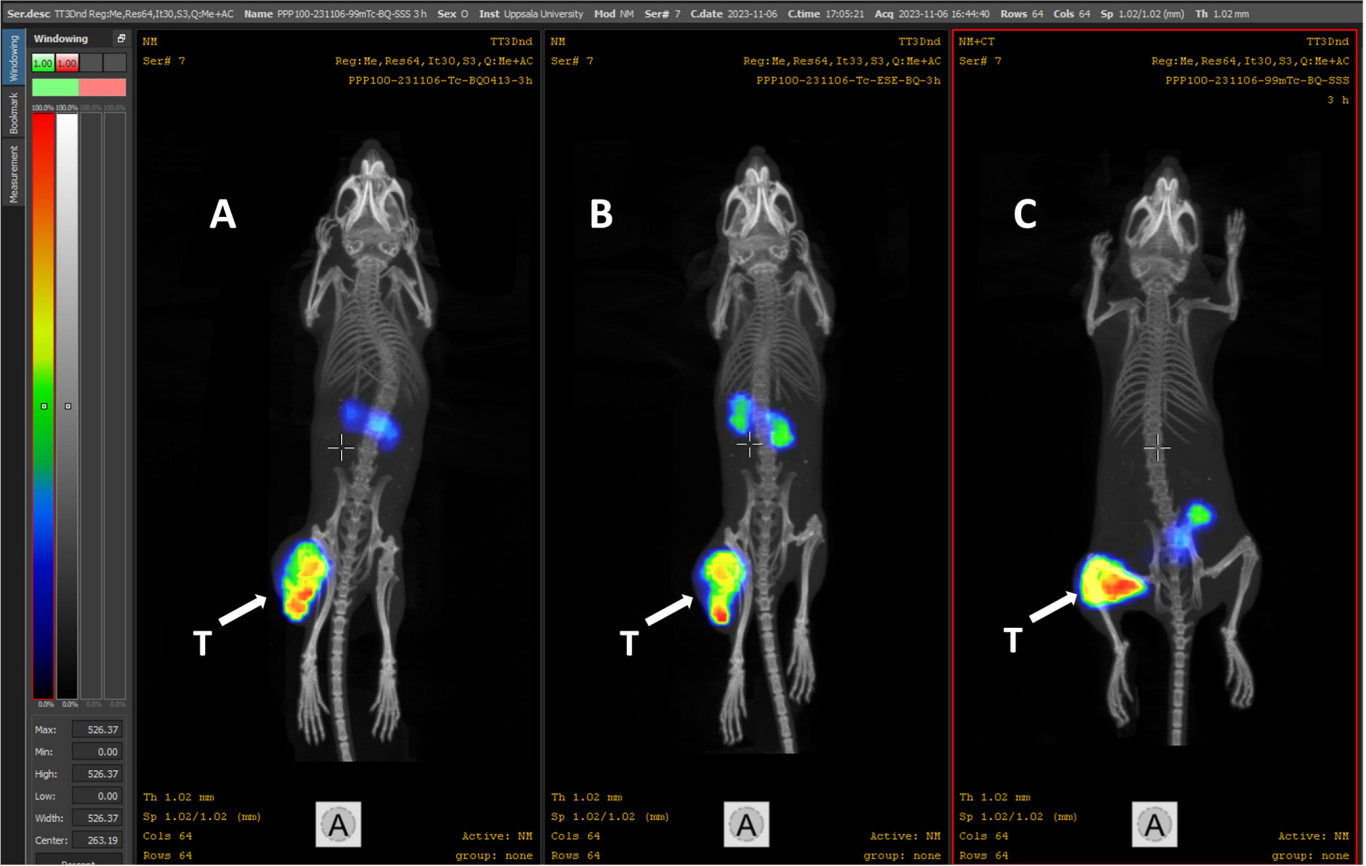
Representative nanoScan SPECT/CT images of PC3-pip xenografted mice injected with 5 nmol (1 MBq) [^99m^Tc]­Tc-BQ0413 (A), [^99m^Tc]­Tc-BQ0500 (B), and [^99m^Tc]­Tc-BQ0501 (C) at 3 h pi. The linear scale was adjusted to the first red pixel in the tumor. White arrows point to tumors (T).

## Discussion

Over the past decade, there have been dramatic improvements in the detection and staging of PCa with the widespread introduction of PSMA-targeting agents based on the EuK binding moiety in clinical practice.^30,31^ However, the majority of the PSMA-targeting tracers were developed for PET.^32^ Because of the decline in SPECT research programs due to the development of new PET tracers, the emergence of new technetium-99m tracers is rare.^13,33^ However, technetium-99m remains the preferred nuclide for SPECT imaging due to its physical decay properties, widespread availability, and versatile coordination chemistry. It continues to play a crucial role in diagnostic nuclear medicine, as the number of clinical examinations based on technetium-99m surpasses those using PET agents. PSMA-SPECT may serve as a cost-effective and more available alternative to PSMA–PET.^13,33^

In the present study, we further evaluated the effect of various mercaptoacetyl-based chelators on the pharmacokinetics and tumor targeting of technetium-99m-labeled PSMA-directed agents, aiming to optimize the biodistribution profile for further clinical translation. The earlier developed PSMA-targeting tracer, BQ0413, with affinity in a low picomolar range, demonstrated sufficient tumor-targeting properties and favorable dosimetry both in a preclinical setting and in PCa patients.^14,15^ [^99m^Tc]­Tc-BQ0413 (maE_3_ chelator) exhibited rapid clearance via glomerular filtration. However, it showed long activity retention in the kidneys due to the physiological expression of PSMA.^15^ The exchange from a negatively charged maE_3_ chelator to the maG_3_ chelator in the next-generation tracers BQ0411 and BQ0412 resulted in decreased renal accumulation but led to increased activity uptake in the GI tract, which might limit the detection of PCa metastases in the abdominal area.^21^ As the low retention and optimal clearance of activity from normal tissues ensure high T/O uptake ratios and result in a high imaging contrast,^34^ further optimization of the BQ0413 chelator structure was needed.

The use of mercaptoacetyl-based chelators containing polar uncharged serine has been investigated for different biomolecules, demonstrating promising results as a pharmacokinetic modifier.^23–25^ Moreover, it was shown that a combination of glutamate and serine in the chelating part might reduce retention of activity in the renal tubules due to reduced residualizing properties of the labeled catabolites.^24^ We have now investigated whether the biodistribution of BQ0413 can be modified by the combination or substitution of negatively charged glutamate residues in the chelator with serine, thereby reducing the renal activity retention. Two novel radiotracers were designed, [^99m^Tc]­Tc-BQ0500 with a maESE chelator and [^99m^Tc]­Tc-BQ0501 with a maS_3_ chelator (Figure 1).

The labeling of both tracers with technetium-99m using a preformulated kit approach was equally efficient, and the labeled products were shown to be stable (Figures 2, S1, and S2). The shoulders observed in the radiochromatograms (Figure 2) may represent the presence of minor isomeric forms of the technetium complex, which is a known phenomenon due to the possible coordination variability of technetium. The small additional peaks are likely attributable to trace levels of radiolysis products, which can form over time due to the radioactive decay and are commonly observed in radiopharmaceutical formulations.^26^ The octanol/water distribution proved that the hydrophilicity of the new constructs was the same as for the reference construct BQ0413.^15^ The new tracers demonstrated PSMA-mediated binding in vitro (Figure 3). Internalization of [^99m^Tc]­Tc-BQ0500 and [^99m^Tc]­Tc-BQ0501 (see Figure 4) by PSMA-transfected PC3-pip cells was as efficient as that for [^99m^Tc]­Tc-BQ0413 ^15^ and showed quite a similar profile with a relatively rapid internalization that increased up to 8 h of incubation. The affinity of the new tracers toward PSMA was slightly reduced compared with [^99m^Tc]­Tc-BQ0413, but it was better than for the maG_3_-variants (BQ0411 and BQ0412).^15,21^ However, the picomolar affinity of [^99m^Tc]­Tc-BQ0500 and [^99m^Tc]­Tc-BQ0501, coupled with a rapid internalization, provided good preconditions for efficient PSMA targeting in vivo.

It has been reported that the choice of chelator for technetium-99m labeling of peptides can influence the biodistribution profile, due to their relatively small size.^22–24^ The substitution of a single amino acid in the chelating three-amino-acid sequence could alter the renal activity retention appreciably.^22–24^ In this study, the incorporation of serine(s) into the mercaptoacetyl-based chelator did not result in a shift in the overall hydrophilicity, which means that observed differences in biodistribution should be determined by the overall charge. A biodistribution study was performed in NMRI mice using a 40 pmol/animal tracer to demonstrate the influence of the chelator structure on the pharmacokinetic properties. It was shown previously that 40 pmol/animal injected mass does not block the uptake in normal PSMA-expressing organs and tissues such as kidneys, salivary gland, and spleen.^15^ All three tested tracers demonstrated a major clearance through glomerular filtration. The results from the biodistribution study for the new inhibitors did not show any signs of instability in vivo, such as elevated uptake in the tail organs for free pertechnetate, stomach, and salivary glands. Major chelator-related differences in the biodistribution were observed for [^99m^Tc]­Tc-BQ0501 with a full substitution of glutamates by serines, particularly in kidneys, intestines with content, and carcass (see Table 2). [^99m^Tc]­Tc-BQ0501 demonstrated the lowest kidney activity uptake among the tested tracers at 3 h pi. The most efficient activity washout from the kidneys (between 3 and 24 h pi) was also found for [^99m^Tc]­Tc-BQ0501 (maS_3_)—71-fold decrease, while washout decreased with the increase of negative charge in the chelator: for [^99m^Tc]­Tc-BQ0500 (maESE)—7-fold decrease, and [^99m^Tc]­Tc-BQ0413 (maE_3_)—4.3-fold decrease. The slightly elevated uptake of activity in the gastrointestinal tract for [^99m^Tc]­Tc-BQ0501 might indicate partial hepatobiliary excretion. However, it did not result in an elevated uptake of activity in the liver (see Table 2), which could reflect the low residualizing properties of the ^99m^Tc-maS_3_ complex. [^99m^Tc]­Tc-BQ0501 showed the lowest uptake in the salivary gland and spleen, organs that physiologically express PSMA. These findings prove our hypothesis that incorporation of serine in the chelator might particularly decrease activity retention in normal organs, keeping the optimal hydrophilicity for renal clearance. As a result, the maS_3_ chelator provided improved clearance from nontarget tissues, which correlated with reduced overall charge. This phenomenon can be attributed to the lower residualizing properties of the ^99m^Tc-maS_3_ complex in comparison with ^99m^Tc-maESE and ^99m^Tc-maE_3_. Our results clearly indicate that the charge of the chelator is essential for uptake and retention in excretory organs, although the exact molecular mechanism underlying the improved clearance of ^99m^Tc-maS_3_ from normal organs, including the kidneys, is unknown.

Tumor-targeting capacity was studied in PC3-pip (PSMA-positive) and PC-3 (PSMA-negative) tumor-bearing mice using 5 nmol/animal mass, which permitted reduced normal organ activity uptake, including PSMA-expressing organs, as was demonstrated in our earlier studies.^15^ The uptake of [^99m^Tc]­Tc-BQ0500 and [^99m^Tc]­Tc-BQ0501 was 2 orders of magnitude higher in PSMA-positive PC3-pip than in PSMA-negative PC-3 xenografts, which demonstrated that tumor uptake of both constructs was PSMA-specific (see Figure 6). The higher affinity of [^99m^Tc]­Tc-BQ0413 as a reference among tested constructs did not result in higher tumor accumulation as compared to serine-containing constructs (Table 3). This could be due to the better affinity of [^99m^Tc]­Tc-BQ0413 that prevents deep penetration into tumors due to the binding site barrier. We cannot also exclude that the tumor uptake for [^99m^Tc]­Tc-BQ0413 might be partially blocked by the used peptide mass and that different injected masses would be optimal for tracers with different affinities.

It should be mentioned that the activity uptake in normal PSMA-expressing organs in NMRI mice was higher due to the lower mass used for estimation of the differences in pharmacokinetic profiles of the tested tracers.^15^ The tumor-targeting of serine-containing tracers was efficient and did not show a significant difference between the peptides in [^99m^Tc]­Tc-BQ0500 and [^99m^Tc]­Tc-BQ0501. Compared to the reference, the new tracers showed decreased salivary gland and bone activity uptake at both 1 and 3 h pi. The blockable activity uptake in salivary glands is consistent with PSMA-specific binding and not tracer instability. Regarding the activity uptake in kidneys, [^99m^Tc]­Tc-BQ0501 had the lowest uptake at 3 h pi among the tested tracers, being 2.3-fold lower compared to [^99m^Tc]­Tc-BQ0413 and 8.3-fold lower compared to [^99m^Tc]­Tc-BQ0500. Both [^99m^Tc]­Tc-BQ0413 and [^99m^Tc]­Tc-BQ0501 had rapid washout from kidneys between 1 and 3 h pi, activity uptake decreased 61% and 81%, respectively, compared to 35% for [^99m^Tc]­Tc-BQ0500. Similar to the biodistribution profile in NMRI mice, [^99m^Tc]­Tc-BQ0501 exhibited significantly higher uptake of activity in the gastrointestinal tract (with content). This might hamper the detection of abdominal metastases shortly after administration and thus delay imaging. Interestingly, the same feature (elevated background activity in the bowel) was shown for another EuK-based PSMA inhibitor containing the maS_3_ chelator—[^99m^Tc]­Tc-PSMA-I&S.^35^ Slow whole-body clearance and, in part, hepatobiliary excretion of [^99m^Tc]­Tc-PSMA-I&S was assumed to be caused by high plasma protein binding and increased lipophilicity.^25^ It is hard to directly compare the hydrophilicity of [^99m^Tc]­Tc-PSMA-I&S and our tracers, as the determination of octanol/water distribution could be performed under different experimental conditions, which were not described in detail. However, the octanol/water distribution coefficients of [^99m^Tc]­Tc-BQ0500 and [^99m^Tc]­Tc-BQ0501 were comparable to that of [^99m^Tc]­Tc-PSMA-I&S (−3.0).^25^ The comparison of the published in vivo biodistribution data for [^99m^Tc]­Tc-PSMA-I&S with our results is not easy due to the different injected tracers’ masses (5 nmol in this study and 0.1 nmol for [^99m^Tc]­Tc-PSMA-I&S) and the different models used (LnCAP tumor-bearing CB-17 severe combined immunodeficiency mice for [^99m^Tc]­Tc-PSMA-I&S).^25^ However, the activity uptake for [^99m^Tc]­Tc-PSMA-I&S in blood, lungs, liver, and muscles was, in general, similar to our tracers. [^99m^Tc]­Tc-BQ0500, [^99m^Tc]­Tc-BQ0501, and [^99m^Tc]­Tc-BQ0413 demonstrated appreciably lower activity uptake in spleen, stomach, intestine, and kidneys compared to [^99m^Tc]­Tc-PSMA-I&S.^25^ The 4–6-fold higher activity uptake in the kidneys and 20-fold higher uptake in the spleen for [^99m^Tc]­Tc-PSMA-I&S could be attributed to a 50-fold lower tracer’s injected mass, while elevated activity uptake in the stomach and intestines could be evidence of label instability.^36^ It is important to note that the chemical structure of PSMA-I&S differs from that of our tracers. While there are certain similarities—such as the presence of the Glu-urea-Lys binding motif, aromatic functional groups within the linker, and a serine-containing chelator—there are also key differences. The most notable distinctions lie in the length of the linker and the positioning of functional groups, both of which significantly influence binding characteristics. In our case, the linker structure was previously optimized to enhance affinity through additional interactions within PSMA subpocket 2, resulting in improved binding properties.^37^ Furthermore, PSMA-I&S incorporates d-serines in its chelating moiety, whereas our tracers utilize l-serines, which may also impact coordination chemistry and overall biodistribution.^36^

[^99m^Tc]­Tc-BQ0500, [^99m^Tc]­Tc-BQ0501, and [^99m^Tc]­Tc-BQ0413 had similar hydrophilicity according to Log *D* values, but the activity concentration in blood was 1.5-fold higher for [^99m^Tc]­Tc-BQ0501 at 1 h pi. This resulted in higher tumor uptake for [^99m^Tc]­Tc-BQ0501 at 3 h of pi, contributing to overall higher T/O ratios, which might translate into images with higher contrast (Table 4). The most important T/O ratios were tumor-to-lung, tumor-to-liver, and tumor-to-bone ratios of [^99m^Tc]­Tc-BQ0501 as compared to reference [^99m^Tc]­Tc-BQ0413, since these organs are frequently metastatic sites in PCa.^38^ The acquired SPECT/CT images indicated clear detection of the PSMA-expressing tumors and confirmed the results from ex vivo biodistribution, demonstrating better renal activity clearance but somewhat elevated uptake in the gastrointestinal tract for [^99m^Tc]­Tc-BQ0501 (Figure 7).

Table 5 provides a comparative overview of the characteristics of [^99m^Tc]­Tc-BQ0500 and [^99m^Tc]­Tc-BQ0501 alongside selected previously published technetium-99m-labeled PSMA-targeting tracers.

**Table 5:** Comparison of [^99m^Tc]­Tc-BQ0500 and [^99m^Tc]­Tc-BQ0501 with Previously Published PSMA-Targeting Tracers Labeled with Technetium-99m

tracer	xenografts	*K*_D_/IC_50_	tumor uptake (time point)	tumor-to-blood ratio (time point)	excretion pathway	reference
[^99m^Tc]Tc-BQ0500	PC3-pip	*K*_D1_ = 0.3 nM	31 ± 4%IA/g (3 h)	109 ± 21 (3 h)	predominantly renal	
		*K*_D2_ = 0.05 nM				
[^99m^Tc]Tc-BQ0501	PC3-pip	*K*_D1_ = 0.5 nM	35 ± 4%IA/g (3 h)	299 ± 160 (3 h)	predominantly renal	
		*K*_D2_ = 3.6 nM				
[^99m^Tc]Tc-BQ0413	PC3-pip	*K*_D_ = 33 ± 15 pM	33 ± 3%IA/g (3 h)	314 ± 73 (3 h)	renal	^15^
[^99m^Tc]Tc-PSMA-I&S	LnCAP	IC_50_ = 40 ± 0.4 nM	8 ± 3%ID/g (1 h)	≈4.9 (1 h)^a^	predominantly renal	^36^
[^99m^Tc]Tc-MIP-1404	LnCAP	*K*_D_ = 1.1 ± 0.9 nM	11 ± 4%ID/g (4 h)	550 (4 h)	renal	^12^
[^99m^Tc]Tc-MIP-1405	LnCAP	*K*_D_ = 4.3 ± 0.3 nM	9 ± 2%ID/g (4 h)	33 (4 h)	renal	^12^
[^99m^Tc]Tc-MIP-1427	LnCAP	*K*_D_ = 0.6 ± 0.4 nM	8 ± 1%ID/g (4 h)	29 (4 h)	renal	^12^
[^99m^Tc]Tc-MIP-1428	LnCAP	*K*_D_ = 1.8 ± 0.3 nM	7 ± 3%ID/g (4 h)	51 (4 h)	renal	^12^
[^99m^Tc]Tc-HYNIC-PSMA	LnCAP	not reported	19.45 ± 2.14%ID/g (2 h)	24 (2 h)	renal	^39^
[^99m^Tc]Tc-PSMA-T4	LnCAP	*K*_D_ = 5.4 ± 2.3 nM	21 ± 4%ID/g (4 h)	≈162 (4 h)^a^	renal	^40^

a The value was calculated based on the data presented in the original research article.

A comparison of the performance of [^99m^Tc]­Tc-BQ0500 and [^99m^Tc]­Tc-BQ0501 with previously reported technetium-99m-labeled PSMA-targeting tracers (Table 5) indicates that our compounds exhibit comparable or superior PSMA binding affinity. While direct comparison of absolute tumor uptake values is limited by the use of different in vivo models (LnCAP and PC3-pip), both BQ0500 and BQ0501 demonstrate high tumor accumulation as early as 1 h pi. Notably, they also show high tumor-to-blood ratios, which suggest enhanced imaging contrast and the potential for improved diagnostic performance in clinical settings. Taken together, the biodistribution profile and tumor targeting properties of technetium-99m-labeled PSMA-targeting tracers can be influenced significantly by the modification of the chelator structure. Besides tumor uptake, fast background clearance from blood and critical organs typically correlates with higher imaging contrasts. In this study, full replacement of glutamate residues with serines resulted in improved overall clearance and favorable tumor-to-background ratios, which might have a high impact on image quality. At the same time, the introduction of serine residues slightly increased the level of activity in the gastrointestinal tract, which might lead to false-positive findings because of occasional formation of hot spots. Since an optimal clearance pattern permits detection of abdominal metastases, a reduction of activity in the gastrointestinal tract is crucial to further improve the imaging properties of [^99m^Tc]­Tc-BQ0501 as a promising probe for SPECT diagnostic imaging in PCa patients.

## Conclusion

We developed two novel EuK-based PSMA-targeting tracers, [^99m^Tc]­Tc-BQ0500 and [^99m^Tc]­Tc-BQ0501, for visualization of PCa using SPECT, both with picomolar affinity to PSMA. These newly developed tracers were evaluated in vitro and in vivo in comparison with the previously developed tracer [^99m^Tc]­Tc-BQ0413. The present study demonstrates that a modification of the chelator (that does not participate in PSMA-binding) plays a critical role in the tumor targeting and pharmacokinetic properties for EuK-based tracers. Specifically, replacement of the maE_3_ chelator by the maS_3_ chelator reduced renal activity retention and improved tumor-to-background ratios while moderately increasing abdominal activity uptake.

## Supplementary Material



## Notes

Radio-HPLC chromatograms demonstrating the stability of [^99m^Tc]­Tc-BQ0500 and [^99m^Tc]­Tc-BQ0501 and in vivo targeting specificity of [^99m^Tc]­Tc-BQ0500 and [^99m^Tc]­Tc-BQ0501 (PDF)
