# Supplementary material for: Reduction of Negative Charge in Mercaptoacetyl-Based Chelators Influences the Biodistribution of Prostate-Specific Membrane Antigen-Targeting Pseudopeptides Labeled with Technetium-99m
Source: ACS Pharmacol Transl Sci. 2025 Sep 25;8(10):3600–12. doi: 10.1021/acsptsci.5c00428 (PMC12519282; doi:10.1021/acsptsci.5c00428)
Supplement: Supplementary file 1 [file pt5c00428_si_001.pdf]

Supporting Information

The reduction of negative charge in mercaptoacetyl-based chelators influences the biodistribution of Prostate Specific Membrane Antigen (PSMA)-targeting pseudo-peptides labeled with technetium-99m

Ekaterina Bezverkhniaia [1,2,*], *Panagiotis Kanellopoulos* [1], *Ulrika Rosenström* [1], *Vladimir Tolmachev* [2,3], *Anna Orlova* [1,4]

[1] Department of Medicinal Chemistry, Uppsala University, 751 23 Uppsala, Sweden; ekaterina.bezverkhniaia@ilk.uu.se (E.B.); panagiotis.kanellopoulos@ilk.uu.se (P.K.); ulrika.rosenstrom@ilk.uu.se (U.R.); anna.orlova@ilk.uu.se (A.O.)

[2] Research Center for Oncotheranostics, Research School of Chemistry and Applied Biomedical Sciences, Tomsk Polytechnic University, 634009 Tomsk, Russia

[3] Department of Immunology, Genetics and Pathology, Uppsala University, 752 37 Uppsala, Sweden; vladimir.tolmachev@igp.uu.se (V.T.)

[4] Science for Life Laboratory, Uppsala University, 752 37 Uppsala, Sweden

***Corresponding author:** Ekaterina Bezverkhniaia, Email: ekaterina.bezverkhniaia@ilk.uu.se

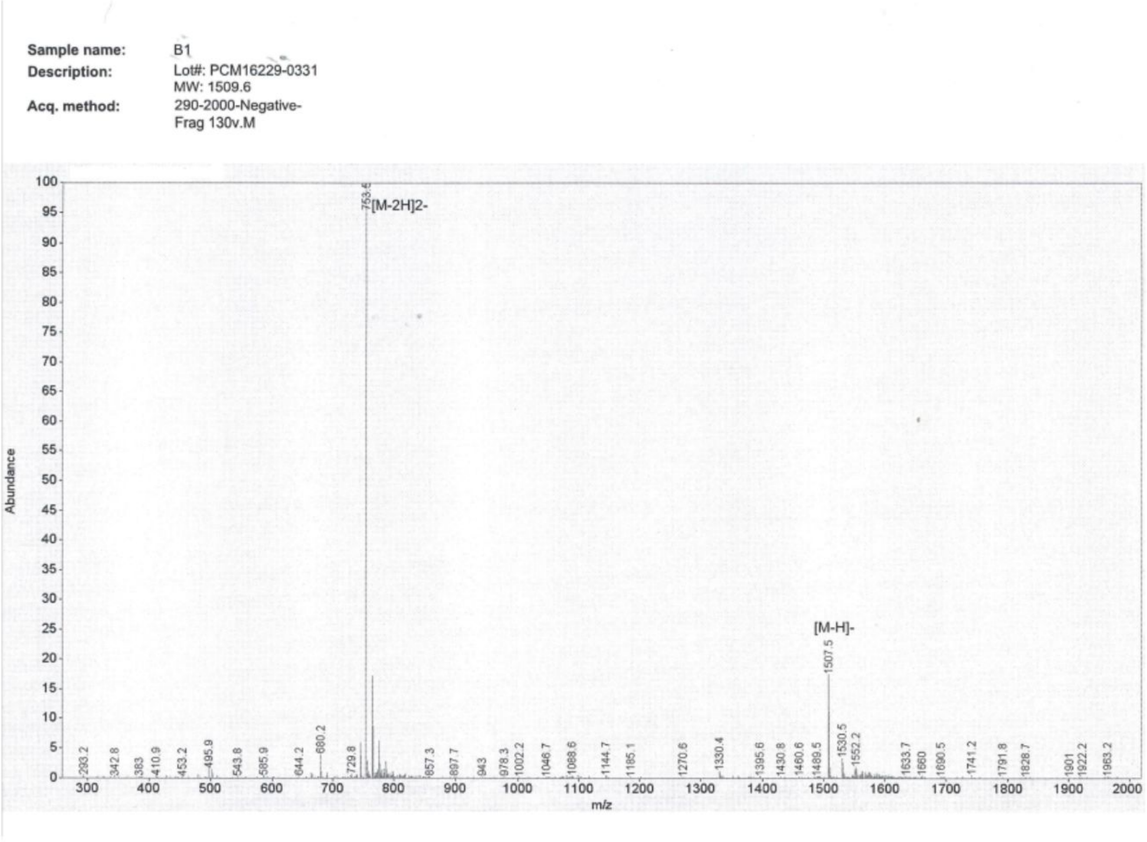

Figure S1. Mass spectrum of BQ0413 provided by Pepmic Co., Ltd. (Suzhou, China).

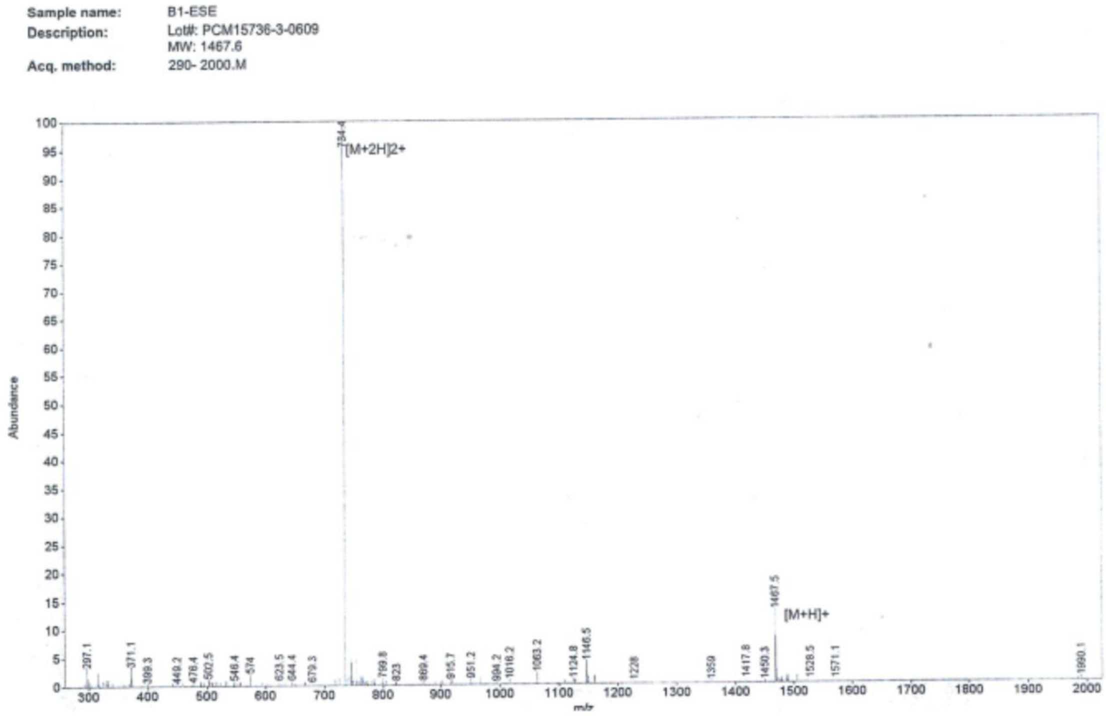

Figure S2. Mass spectrum of BQ0500 provided by Pepmic Co., Ltd. (Suzhou, China).

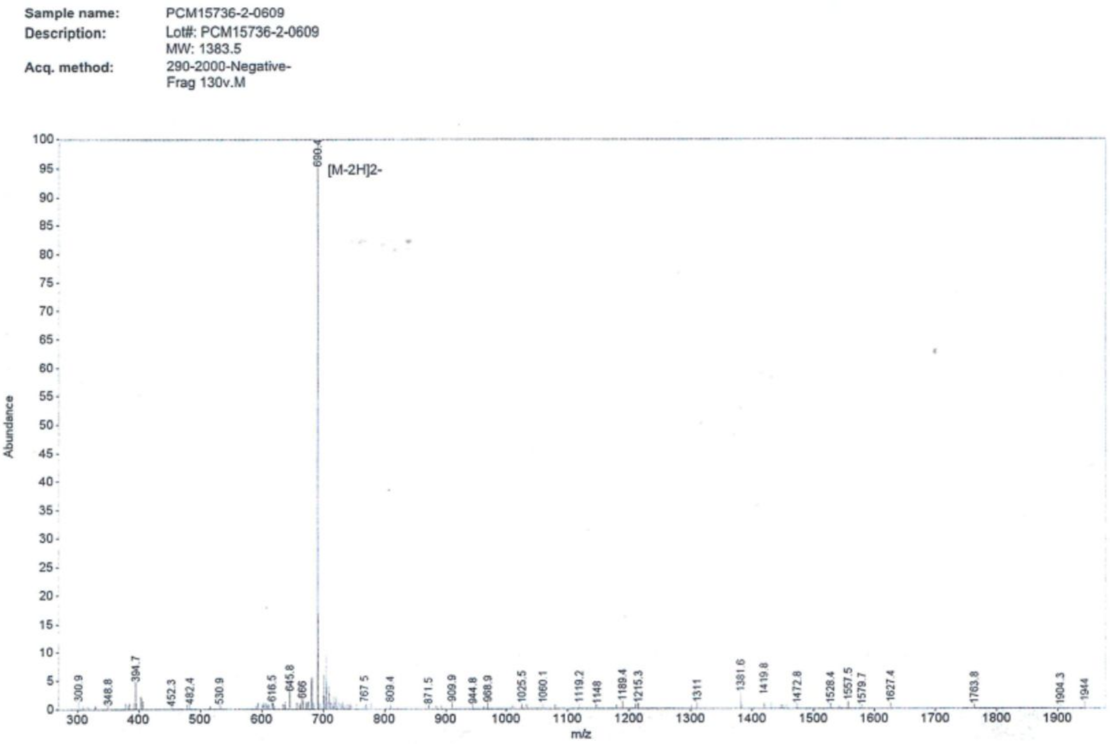

Figure S3. Mass spectrum of BQ0501 provided by Pepmic Co., Ltd. (Suzhou, China).

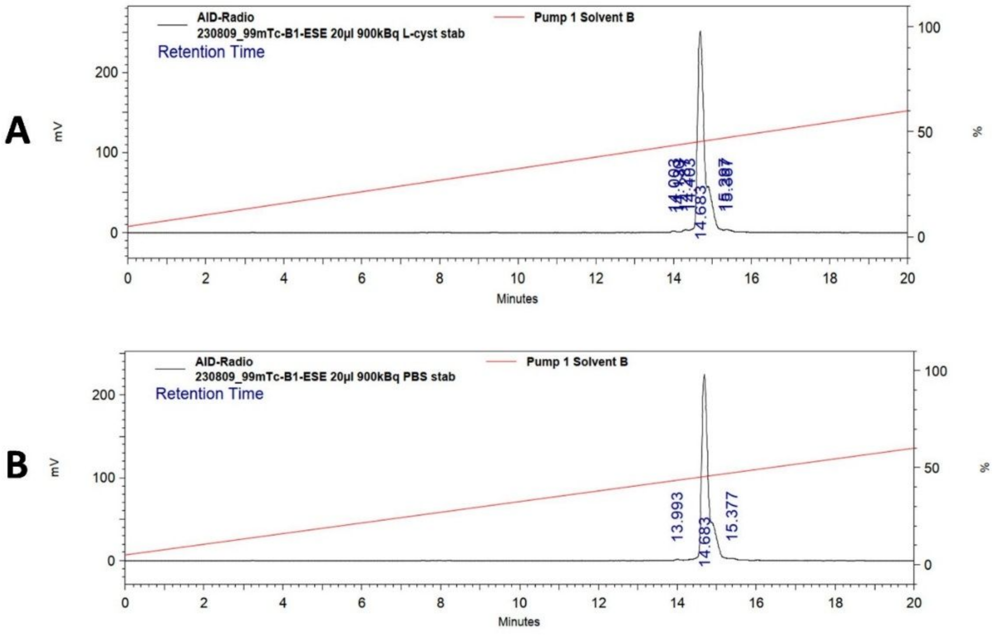

Figure S4. Radio-HPLC chromatogram of [^{99m}Tc]Tc-BQ0500 showing stability in the presence of a 300-fold molar excess of L-cysteine (**A**) and PBS (**B**) after 1h incubation at room

temperature. The reference chromatogram at t = 0 min for the stability studies corresponds to the radio-HPLC chromatogram of the freshly prepared labeling solution, which is presented in Figure 2 of the main manuscript. Radio-HPLC analysis was performed using a Hitachi Chromaster HPLC system with a radioactivity detector and Phenomenex Luna® C18 column (100 Å; 150×4.6 mm; 5 μm) at room temperature (20◦C). Solvent A was 0.1% trifluoroacetic acid (TFA) in H2O, solvent B was 0.1% TFA in acetonitrile, and the flow rate was 1 mL/min. For identity and purity analysis, the method with a gradient from 5 to 60% solvent B over 20 min was used.

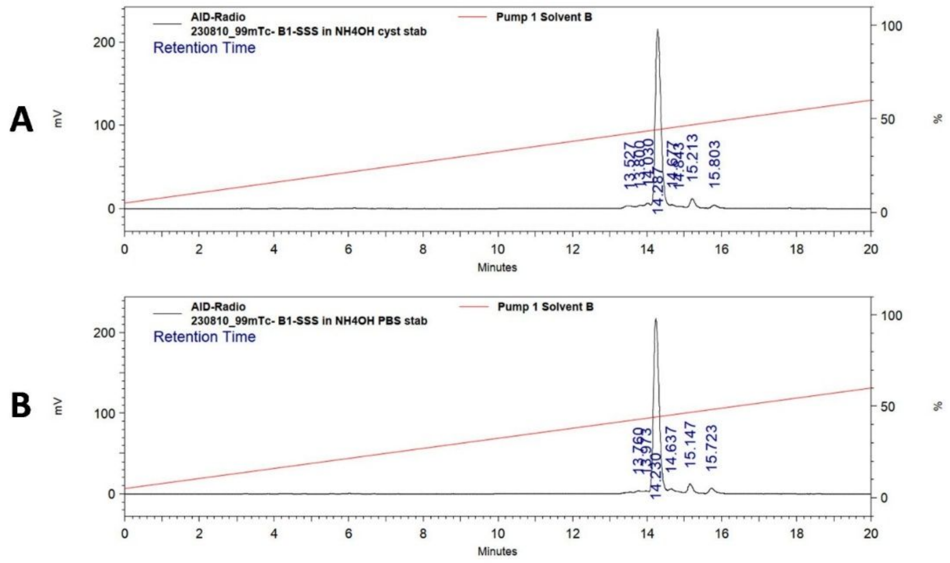

Figure S5. Radio-HPLC chromatogram of [^{99m}Tc]Tc-BQ0501 showing stability in the presence of a 300-fold molar excess of L-cysteine (**A**) and PBS (**B**) after 1h incubation at room temperature. The reference chromatogram at t = 0 min for the stability studies corresponds to the radio-HPLC chromatogram of the freshly prepared labeling solution, which is presented in Figure 2 of the main manuscript. Radio-HPLC analysis was performed using a Hitachi Chromaster HPLC system with a radioactivity detector and Phenomenex Luna® C18 column (100 Å; 150×4.6 mm; 5 μm) at room temperature (20◦C). Solvent A was 0.1% trifluoroacetic acid (TFA) in H2O, solvent B was 0.1% TFA in acetonitrile, and the flow rate was 1 mL/min.

For identity and purity analysis, the method with a gradient from 5 to 60% solvent B over 20 min was used.

Table S1. In vivo targeting specificity of [^{99m}Tc]Tc-BQ0500 and [^{99m}Tc]Tc-BQ0501 after injection of 5 nmol (60 kBq, the mass of the injected compound was adjusted with unlabeled tracer and injected in a volume of 100 µl of 1% BSA in PBS) in PSMA-positive PC3-pip and PSMA-negative PC-3 tumor-bearing mice 3 h post-injection. Data are expressed as the percentage of administered activity per gram of tissue (% IA/g). The data are presented as the average (n = 4) and SD.

**Organ**	**[^{99m}Tc]Tc-BQ0500**		**[^{99m}Tc]Tc-BQ0501**	
	**PC3-pip (PSMA+)**	**PC-3 (PSMA-)**	**PC3-pip (PSMA+)**	**PC-3 (PSMA-)**
Blood	0.3±0.1	0.25±0.07	0.14±0.07	0.14±0.07
Salivary	0.2±0.1	0.24±0.07	0.14±0.06	0.11±0.05
Lung	0.4±0.1	0.33±0.06	0.21±0.07	0.19±0.07
Liver	0.4±0.1	0.6±0.3	0.18±0.06	0.15±0.06
Spleen	1.4±0.3 *	0.8±0.2	0.3±0.2	0.27±0.19
Pancreas	0.3±0.2	0.3±0.2	0.1±0.1	0.1±0.1
Stomach	1.6±1.2	0.25±0.05	0.14±0.06	0.11±0.06
Small intestines	0.4±0.2	0.7±0.4	0.17±0.03	0.14±0.07
Kidney	33±3 *	24±3	4±2	4.1±0.7
Tumor	31±4 *	0.36±0.04	35±4 *	0.12±0.02
Muscle	0.10±0.03	0.08±0.03	0.08±0.04	0.04±0.01

Bone	0.10±0.02	0.10±0.01	0.07±0.03	0.04±0.01
Intestines with content	5.4±0.3	6.2±0.8	11.0±0.5	10.3±0.8
Carcass	4±1	2.2±0.8	3±1*	1.2±0.6

*Significant difference ($p < 0.05$ in 2-tailed t-test) between PC3-pip (PSMA+) and PC-3 (PSMA-) for corresponding tracer.
